# Plasma Corticosterone Activates SGK1 and Induces Morphological Changes in Oligodendrocytes in Corpus Callosum

**DOI:** 10.1371/journal.pone.0019859

**Published:** 2011-05-31

**Authors:** Shingo Miyata, Yoshihisa Koyama, Kana Takemoto, Keiko Yoshikawa, Toshiko Ishikawa, Manabu Taniguchi, Kiyoshi Inoue, Miwa Aoki, Osamu Hori, Taiichi Katayama, Masaya Tohyama

**Affiliations:** 1 Department of Anatomy and Neuroscience, Graduate School of Medicine, Osaka University, Suita, Osaka, Japan; 2 Department of Clinical Disorder Research, The Osaka-Hamamatsu Joint Research Center For Child Mental Development, Suita, Osaka, Japan; 3 Center for Behavioral Neuroscience, Yerkes National Primate Research Center, Emory University, Atlanta, Georgia, United States of America; 4 Department of Neuroanatomy, Graduate School of Medical Science, Kanazawa University, Kanazawa, Ishikawa, Japan; 5 Department of Child Development and Molecular Brain Science, United Graduate School of Child Development, Osaka University, Kanazawa University and Hamamatsu University School of Medicine, Suita, Osaka, Japan; Rikagaku Kenkyūsho Brain Science Institute, Japan

## Abstract

Repeated stressful events are known to be associated with onset of depression. Further, stress activates the hypothalamic–pituitary–adrenocortical (HPA) system by elevating plasma cortisol levels. However, little is known about the related downstream molecular pathway. In this study, by using repeated water-immersion and restraint stress (WIRS) as a stressor for mice, we attempted to elucidate the molecular pathway induced by elevated plasma corticosterone levels. We observed the following effects both, *in vivo* and *in vitro*: (1) repeated exposure to WIRS activates the 3-phosphoinositide-dependent protein kinase (PDK1)–serum glucocorticoid regulated kinase (SGK1)–*N*-myc downstream-regulated gene 1 (NDRG1)–adhesion molecule (i.e., *N*-cadherin, α-catenin, and β-catenin) stabilization pathway *via* an increase in plasma corticosterone levels; (2) the activation of this signaling pathway induces morphological changes in oligodendrocytes; and (3) after recovery from chronic stress, the abnormal arborization of oligodendrocytes and depression-like symptoms return to the control levels. Our data strongly suggest that these abnornalities of oligodendrocytes are possibly related to depression-like symptoms.

## Introduction

Major depression is thought to be a multifactorial disease related to both environmental and genetic factors. However, the genes responsible and the pathogenesis of major depression at the molecular level remain unclear. Among many environmental factors, repeated stressful events are associated with the onset of depression, and stress activates the hypothalamic–pituitary–adrenocortical (HPA) system [1]–[5].

The HPA system is initiated by the activation of the paraventricular nucleus of the hypothalamus, leading to the secretion of corticotropin-releasing hormone from the neuron terminals of the paraventricular nucleus. Corticotropin-releasing hormone triggers the release of adrenocorticotropic hormone from the anterior pituitary. Adrenocorticotropic hormone subsequently stimulates the release of cortisol or corticosterone in humans and rodents, respectively. It is reported that the negative feedback of corticosteroids on the HPA system occurs at the level of the hypothalamus and the anterior pituitary *via* the glucocorticoid receptors [6].

Dysregulation of this negative feedback mechanism is reported in patients with major depressive disease, which results in hyperactivity of the HPA axis and higher basal levels of serum corticosterone [7], [8]. Antidepressant treatment partly normalizes the hyperactivity of the HPA axis in depressed patients [9]. In addition, many clinical cases demonstrate that elevated corticosterone levels trigger depressive symptoms. For example, patients with Cushing disease, in whom corticosteroids are excessively secreted, frequently exhibit depressive symptoms; patients chronically treated with exogenous corticosteroids exhibit express depressive symptoms referred to as steroid psychosis [10]. These facts strongly indicate that sustained elevated levels of plasma corticosteroids are one of the causes of major depressive diseases.

However, the molecular pathway in the brain affected by excess levels of plasma corticosteroids is not known. Here, we used water-immersion restraint stress (WIRS) as a stressor and demonstrated that chronically elevated plasma corticosterone levels induce the upregulation of adhesion molecules such as *N*-cadherin, α-catenin, and β-catenin in the oligodendrocytes *via* the activation of phosphatidylinositol 3-kinase (PI3K)–3-phosphoinositide-dependent protein kinase (PDK1)–serum glucocorticoid regulated kinase (SGK1)–*N*-myc downstream-regulated gene 1 (NDRG1) pathway, resulting in morphological changes in the oligodendrocytes.

## Materials and Methods

### Ethics Statement

All animal care and handling procedures were approved by the Institutional Animal Care and Use Committee of Osaka University (No. 19-041-02), the Guiding Principles for the Care and follow the United States National Institutes of Health Guide for the Care and Use of Laboratory Animals.

### Animals

Naive adult male C57/BL6 mice weighing 25–35 g, 11 weeks old, were obtained from Japan SLC Inc (Hamamatsu, Japan). Three mice per cage were housed in a temperature- (22±2°C), humidity- (55±10%), and light- (12 h light/dark schedule; lights on at 7:00 and off at 19:00 hr) controlled environment and fed laboratory food and water ad libitum. Mice were allowed to adjust to the experimental environment for 1 week before the experiments were performed.

### Stress exposure

The mice were placed in a 50 mL conical polypropylene centrifuge tube and immersed vertically to the level of the xiphoid process into a water bath at 23°C for 2 hrs (acute stress). The mice were subjected to this stress session once a day for 3 weeks (chronic stress). In our preliminary experiments, gastric ulcer was not produced by single or chronic exposure. Control mice were similarly fasted, followed by removal from their home cages, and subsequent placement in new breeding cages for 2 hrs. These experimental groups were chosen by means of a completely randomized design. Depression-like behaviors were measured with tail-suspension test and forced-swimming test. Immobility time was recorded in both tests during the last 6 min in the total 10 min test-period. After the end of this stress session, the mice were anesthetized with administration of sodium pentobarbital (30 mg/kg). In chronic stress and control mice groups, to avoid the acute influence of the last stress session and to evaluate the influence of chronic stress as a consequence of the cumulative stress effects, mice were sacrified after 1 day from last stress session.

### Adrenalectomy

Adrenal glands were surgically removed under anesthesia. Sham mice were treated similarly with the exception of the removal of the adrenal glands. Adrenalectomized mice were given 0.7% NaCl in the drinking water to maintain their mineral balance and allowed to recover for 21 days before start of the experiments.

### Measurement of plasma corticosterone levels

After deep anesthesia of all mice at the end of the stress experiments, blood samples were collected into tubes containing heparin between 11:00 and 13:00 h by cardiac puncture. The tubes were immediately placed on ice and then centrifuged at 1,000 *g* for 15 min at 4°C. Plasma was stored at −80°C prior to the enzyme immunoassay. Plasma corticosterone levels were determined in duplicate using a Corticosterone enzyme immune assay kit (Cayman Chemical Comp., Ann Arbor, MI, USA).

### Dexamethazone (DEX) administration

Mice were intraperitoneally injected with dexamethazone (3 mg/kg; dexamethasone 21-phosphate disodium salt, Sigma Chemical Co., St. Louis, MO) dissolved in saline. Control animals were always given an appropriate vehicle treatment.

### 5-Bromodeoxyuridine (BrdU) incorporation and BrdU immunostaining

Mice were injected 4 times intraperitoneally with 50 mg/kg BrdU (50 mg/kg; Sigma, St. Louis, MO, USA) at 6 hrs intervals in a day. Mice were perfused with PBS for 3 min and 4% PFA in pH 7.2 PBS for 5 min. Brains were excised and postfixed in 4% PFA for 72 hrs at 4°C, then in 30% sucrose for at least 2 days. Brain sections were incubated in 2 N HCl for 15 min at 37°C, washed in PBS, and incubated in anti-BrdU (primary) antibody (mouse monoclonal 1 ∶ 20; Dako) in PBS containing 5% normal goat serum overnight at 4°C. After washing with PBS, sections were incubated in Alexa488-labeled anti-rat secondary antibody (1 ∶ 500; Invitrogen, USA) for 1 hr at 20°C.

### Reverse transcriptase reaction and real-time polymerase chain reaction (PCR)

Total RNA was prepared from the corpus callosum of stress and control mice groups by using ISOGEN (NipponGene, Toyama, Japan) according to the manufacturer's instructions. The total RNA extract was reverse transcribed by using oligo(dT)12–18 primers and SuperScript III RNaseH reverse transcriptase (Invitrogen Corp.) according to the manufacturer's instructions. Real-time PCR was performed using an ABI PRISM 7900HT Sequence Detection System with the SybrGreen PCR Master Mix (Applied Biosystems, Foster, CA, USA). To quantify the expression levels of *Sgk1* and *Ndrg1*, the following primers were used: *Sgk1* forward primer, 5′-GGGTGCCAAGGATGACTTTA-3′ (complement of bases 1020–1039); *Sgk1* reverse primer, 5′-CTCGGTAAACTCGGGATCAA-3′ (reverse complement of bases 1154–1173); *Ndrg1* forward primer, 5′-CATTTTGCTGTCTGCCATG-3′ (complement of bases 262–280); *Ndrg1* reverse primer, 5′-CCATGCCAATGACACTCTTG-3′ (reverse complement of bases 393–412). Glyceraldehyde-3-phosphate dehydrogenase (*Gapdh*) forward primer 5′-GTGTTCCTACCCCCAATGTG-3′ and *Gapdh* reverse primer 5′-AGGAGACAACCTGGTCCTCA-3′ were used as the initial controls. Sybr Green I fluorescence from the double-stranded PCR products was measured according to the manufacturer's instructions (Applied Biosystems).

### *In situ* hybridization

The partial cDNAs for coding region (CR) of mouse *Sgk1* (nucleotides 357–914), 3′ untranslated region (3′UTR) of mouse *Sgk1* (nucleotides 1449–1805) and mouse *Ndrg1* (nucleotides 315–915) were amplified by RT-PCR using the following primers: CR *Sgk1* forward primer, 5′-AGAAGAAGTATTCTATGCAGC-3′ (complement of bases 357–377); partial CR *Sgk1* reverse primer, 5′-ATCTCAGCCGTGTTCCGGCTA-3′ (reverse complement of bases 894–914); 3′UTR *Sgk1* forward primer, 5′-GAACATTTTAAAAGAATTTGC-3′ (complement of bases 1449–1469); and 3′UTR *Sgk1* reverse primer, 5′-AAACACAAACTGAACACTCTG-3′ (reverse complement of bases 1785–1805); partial *Ndrg1* forward primer, 5′-GGGCAACCGTCCTGTCATCCT-3′ (complement of bases 315–335); partial *Ndrg1* reverse primer, 5′-CACTGCAAAGTGACAGTGTGG-3′ (reverse complement of bases 895–915). The amplified fragments were TA cloned into the pGEM-T vector (Promega Corp.). Serial sagittal and coronal sections that were 14-µm thick were prepared and thaw-mounted on Matsunami Adhesive Silane (MAS)-coated glass slides (Matsunami Glass Ind., Osaka, Japan). The sections were processed for *in situ* hybridization as previously described [11].

### Western blot analysis

Western blot analysis was performed as previously described [12]. See the Materials and Methods S1 for details.

### Plasmid construction

See the Materials and Methods S1 for details.

### Cell culture

Oligodendrocyte primary culture was performed as previously described [13]. See the Materials and Methods S1 for details.

### Immunocytochemical procedure

See the Materials and Methods S1 for details.

### Immunohistochemical procedures

See the Materials and Methods S1 for details.

### Electron microscopy

Mice were anesthetized and killed *via* transcardial perfusion with 4% PFA and 2% glutaraldehyde (vol/vol) in 0.1 M PBS. A block of approximately 1×1×2 mm^3^ was removed from the body of the corpus callosum at the level of the dorsal hippocampus and from the sciatic nerve, incubated for 2 hrs at 4°C in the same fixative, and contrasted with 1% osmic acid (vol/vol) in PBS. Tissues were dehydrated in an ethanol gradient from 50–100% and embedded in Epon. Semi-thin sections (0.9 µm) were stained with toluidine blue for survey by light microscopy. Ultra-thin sections (80 nm) were cut and stained with 2% uranyl acetate (vol/vol, Watson's modified method) and Reynolds lead citrate, and analyzed with a Hitachi H-7650 transmission electron microscope. Non-overlapping digitalized images of fiber cross-sections were obtained and analyzed using Image Pro Plus 3.0 software (Media Cybernetics). The diameter of an axon and myelin thickness was determined from 300–1,000 fibers in the corpus callosum.

## Results

### Simple WIRS (acute stress) increases *Sgk1* expression in fiber tracts

**Figure 1:**
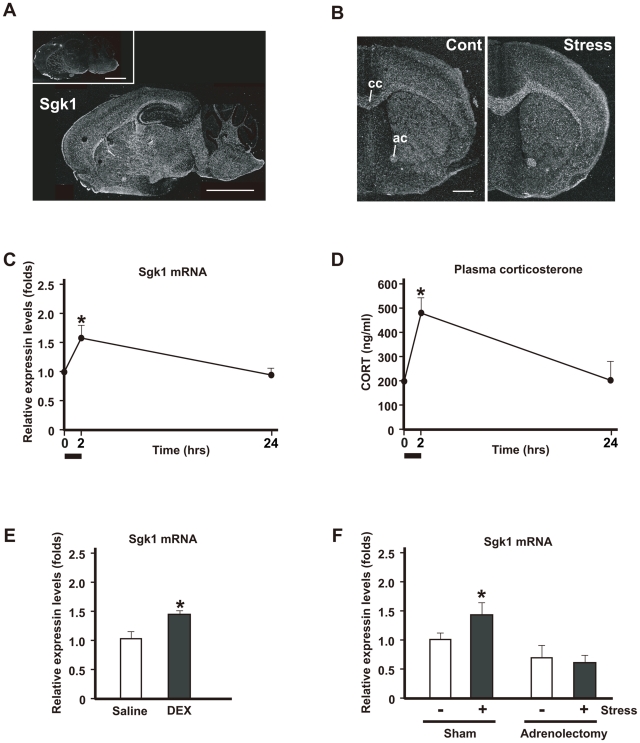
Simple WIRS (acute stress) upregulates *Sgk1* predominantly in the fiber tracts *via* HPA axis activation. (A, B) *In situ* hybridization images of *Sgk1* mRNA. Dark-field photomicrographs showing the distribution of *Sgk1* mRNA-expressing cells in the mouse brain on the sagittal (A) and frontal (B) planes. Sections were hybridized with a ^35^S-labeled antisense RNA probe for *Sgk1* mRNA. As controls, adjacent sections were hybridized with ^35^S-labeled sense RNA probe (inset in a). Scale bar = 5 mm. (B) Simple exposure to WIRS increased *Sgk1* mRNA expression in the mouse brain. cc, corpus callosum; ac, anterior commissure. Scale bar = 2 mm. (C) Relative mRNA expression levels were determined by real-time PCR and normalized to that of GAPDH mRNA. Time-course dependent changes of *Sgk1* mRNA levels in the corpus callosum analyzed after simple exposure to WIRS. *p<0.05, *t*-test. (D) Time-course dependent change of plasma corticosterone levels analyzed by the EIA kit after simple exposure to WIRS. *p<0.05, *t*-test. (E) Relative mRNA expression levels were determined by real-time PCR and normalized to that of GAPDH mRNA. Intraperitoneal administration of 3 mg/kg DEX induced *Sgk1* mRNA expression in the corpus callosum. *p<0.05, *t*-test. (F) Relative mRNA expression levels were determined by real-time PCR and normalized to that of GAPDH mRNA. Adrenorectomy completely inhibited the upregulation of *Sgk1* mRNA after simple exposure to WIRS (compare black bars). *p<0.05, *t*-test.

*In situ* hybridization histochemistry for *Sgk1* mRNA in the brains of normal adult mice shows that two types of cells express *Sgk1* mRNA: those preferentially localized in the fiber tracts (oligodendrocytes) such as the corpus callosum and anterior commissure and the neurons that are localized in the CA3 region of the hippocampal formation (Figure 1A). Both *in situ* hybridization histochemistry and real-time polymerase chain reaction (PCR) for *Sgk1* mRNA detection reveal that *Sgk1* mRNA expression increased markedly in the corpus callosum and the anterior commissure, peaking 2 h following acute stress, and subsequently decreasing to control levels at 24 h (Figure 1B, 1C). However, *Sgk1* transcription in neurons was not affected by acute stress (Figure S1).

Since it is well known that stress activates the HPA axis, we compared the alteration in the plasma corticosterone levels with the time-course expression of *Sgk1* mRNA after exposure to acute stress. The time course of plasma corticosterone levels exhibited a pattern similar to that of *Sgk1* mRNA expression (Figure 1D). To confirm that *Sgk1* mRNA expression depends on plasma corticosterone levels, we examined the effect of the exogenous administration of a synthetic glucocorticoid, dexamethasone (DEX). *Sgk1* mRNA levels in the brain increased after DEX treatment (Figure 1E). In addition, no increase in *Sgk1* mRNA expression was detected in the adrenalectomized mice (Figure 1F).

Thus, simple WIRS (acute stress) increases *Sgk1* mRNA expression in the fiber tracts *via* the HPA axis. Therefore, we subsequently examined whether *Sgk1* mRNA expression is increased in a mouse model of depression-like symptoms wherein the HPA axis plays an important role.

### Exposing mice to repeated WIRS is a suitable model of depression-like symptoms

**Figure 2:**
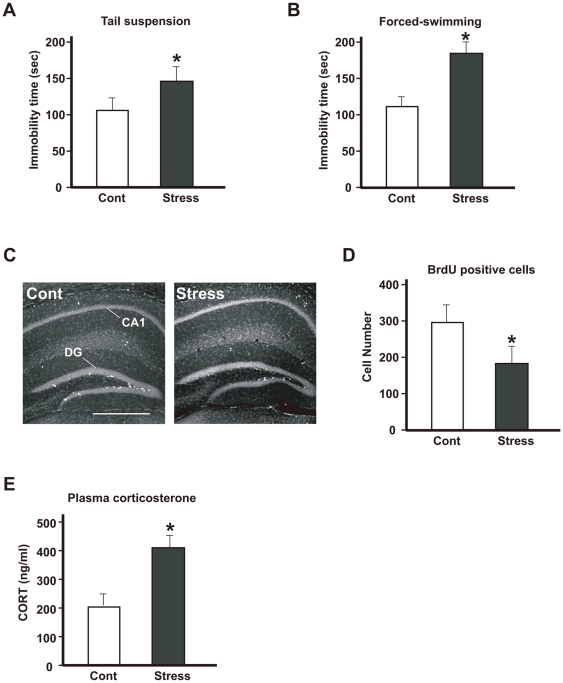
Characterization of model mice exposed to repeated WIRS (chronic stress). (A, B) Effects of repeated WIRS as chronic stress on mouse behavior. Stressed mice exhibited significantly increased depression-like behavior compared with control mice in tail-suspension (A) and forced-swimming (B) tests. The results are expressed as the mean ± SEM of three independent experiments. *p<0.05, *t*-test. (C) Less adult neural stem cells labeled by BrdU were found in the subgranular zone of the dentate gyrus (DG) in the stressed mice (C, Stress) than in the control mice (C, Cont). BrdU (75 mg/kg) was injected 4 times at 6-h intervals. Scale bars = 500 µm. (D) Quantification of the results shown in C. The results are expressed as the mean ± SEM of three independent experiments. *p<0.05, *t*-test. (E) Alternation of plasma corticosterone 24 h after repeated WIRS. The results are expressed as the mean ± SEM of three independent experiments. *p<0.05, *t*-test.

To evaluate the depressive behavior of chronically stressed mice, immobility time was recorded in tail suspension tests during the last 6 min of a 10-min test period. The mice exposed to repeated WIRS (chronic stress) showed significant longer immobility times than control mice, indicating increased despair (Figure 2A). The immobility time recorded in the forced-swimming test was longer in the mice exposed to repeated WIRS (chronic stress) than in the control mice (Figure 2B). In addition, exposing mice to repeated stress resulted in a significant decrease in the number of neural stem cells of the adult hippocampus that took up BrdU (Figure 2C, 2D). Thus, this suggests that chronic stress inhibits neurogenesis in the hippocampus. Furthermore, repeated exposure to WIRS also upregulated of plasma corticosterone levels (Figure 2E).

As demonstrated in the mice exposed to repeated exposure of WIRS, neurogenesis inhibition in the hippocampus, increased immobility time, and continuous upregulation of plasma corticosterone levels are well known to occur in patients with depression, confirming that the present experimental model is suitable for studying depression-like symptoms.

### *Sgk1* mRNA and SGK1 protein expression are upregulated in oligodendrocytes after repeated exposure to WIRS

**Figure 3:**
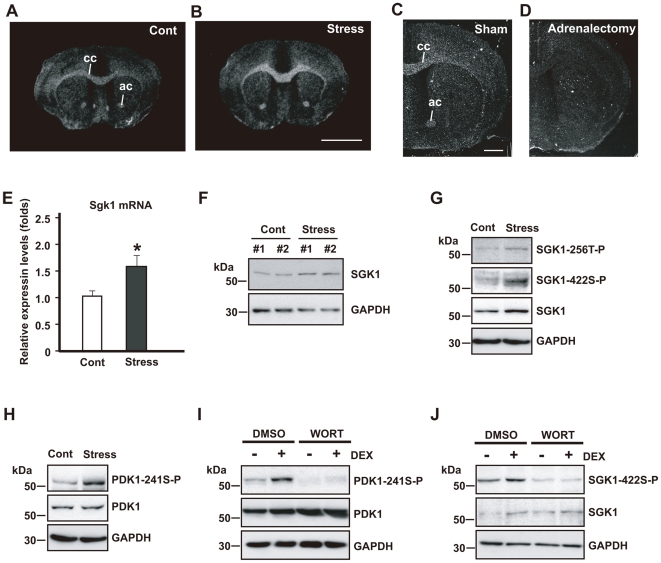
Repeated exposure to WIRS (chronic stress) upregulates SGK1 and SGK1 is activated by PDK1. (A, B) *In situ* hybridization images of *Sgk1* mRNA. Dark-field photomicrographs show the upregulation of *Sgk1* mRNA expression in the fiber tracts after repeated exposure to WIRS (A, controls; B, stress). cc, corpus callosum; ac, anterior commissure. Scale bar = 5 mm. (C, D) *In situ* hybridization images of *Sgk1* mRNA. Upregulation of *Sgk1* mRNA (C) after repeated exposure to WIRS was abolished in the brain of adrenalectomized mice (D). cc, corpus callosum; ac, anterior commissure. Scale bar = 2 mm. (E) Real-time PCR analysis shows a significant increase in *Sgk1* mRNA levels in the corpus callosum of mice exposed to repeated WIRS. *p<0.05, *t*-test. (F) Western blot analysis of SGK1 protein in the control and test (repeated exposure to WIRS) mice. (G, H) Western blot analysis shows SGK1 protein, its phosphorylation at positions T-256 (SGK1-256T-P) and S-422 (SGK1-422S-P), and the phosphorylation of PDK1 at position S-241 (PDK-241S-P) in the oligodendrocytes of the corpus callosum after repeated exposure to WIRS. (I, J) Western blot analysis shows that elevated corticosterone levels upregulate the phosphorylation of PDK1 (I) and SGK1 (J) in HEK293 cells with (+) or without (−) DEX treatment (100 µM). Wortmannin (WORT) was used as an inhibitor of the PI3K signaling pathway.

**Figure 4:**
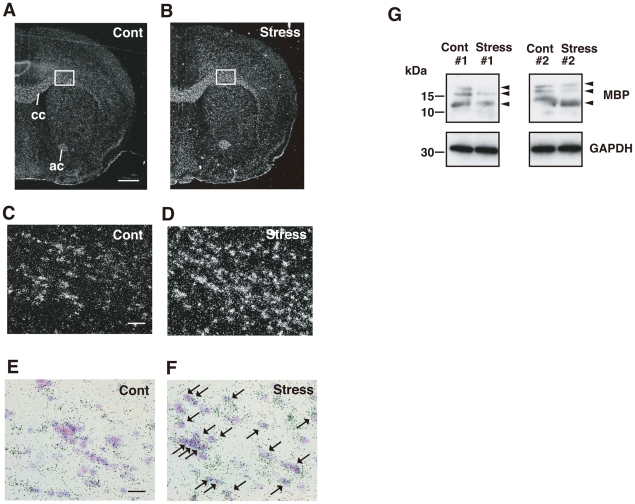
Chronic stress upregulates *Sgk1* predominantly in the fiber tracts *via* HPA axis activation. (A, B) *In situ* hybridization images of *Sgk1* mRNA. *Sgk1* mRNA expression in the corpus callosum (cc) and anterior commissure (ac) was elevated in mice after repeated exposed to WIRS (B) compared with the expression of *Sgk1* mRNA in these bundles (A). Scale bar = 2 mm. (C, D) Enlargement of the squares in (A) and (B), respectively. Scale bar = 100 µm. (E, F) Merged images of Nissl staining and *in situ* hybridization images of *Sgk1* mRNA. The distribution of cells expressing *Sgk1* mRNA in the corpus callosum of the control (E) and repeated WIRS-exposed mice (F) in bright-filed photomicrographs. Positive grains were concentrated in the oligodendrocytes. Scale bars = 50 µm. (G) Western blot analysis shows that repeated WIRS decreases MBP expression in the corpus callosum compared to the control mice.

The *Sgk1* mRNA expression was markedly increased in the fiber tracts such as the corpus callosum and the anterior commissure after repeated exposure to WIRS (Figure 3A, B). *Sgk1* mRNA was almost exclusively localized in oligodendrocytes (Figure 4A–F). In addition, *Sgk1* mRNA upregulation in the fiber tracts after the mice were exposed to repeated WIRS was abolished after adrenolectomy (Figure 3C, D), indicating that elevated levels of corticosterone after exposure to repeated stress increase *Sgk1* expression in the oligodendrocytes. *Sgk1* mRNA and SGK1 protein upregulation in the fiber tracts such as the corpus callosum was also confirmed by quantitative real-time PCR (Figure 3E) and western blotting analysis (Figure 3F), respectively.

### Phosphorylated SGK1 levels are elevated by repetitive exposure to WIRS

We examined the effect of repeated WIRS on the phosphorylation of SGK1, because it is the active form of SGK1 [14]. The activation of SGK1 is known to be dependent upon the phosphorylation of Thr (T)-256 in the activation loop and Ser (S)-422 in the hydrophobic motif near the C terminus [15], [16]. Thus, we examined the phosphorylation levels of 256T and 422S of SGK1 in the corpus callosum after repeated exposure to WIRS. In addition to the increased expression of SGK1, the level of phosphorylated SGK1 was elevated in both sites (Figure 3G).

### SGK1 is activated by activated PDK1, which is phosphorylated by activated PI3K

It has been demonstrated that HEK293 cells phosphorylated PDK1 induced by phosphorylated PI3K in turn phosphorylates SGK1 [14], [16]. Therefore, we examined whether PDK1 phosphorylation levels increase in the corpus callosum after repeated exposure to WIRS.

Chronic stress elevated the level of phosphorylated PDK1. However, no notable alteration was found with respect to PDK1 expression levels (Figure 3H), indicating that exposure to repeated WIRS does not affect PDK1 production but elevates the phosphorylation level of PDK1.

Next, we investigated whether elevated of the plasma corticosterone levels affect SGK1 phosphorylation *via* the activation of the PI3K signaling pathway in HEK293 cells. Stimulating the cells with DEX increased the level of PDK1 phosphorylation; however, the same stimulation did not change the expression level of PDK1 (Figure 3I). Inhibition of the PI3K signaling pathway by wortmannin (WORT) inhibited the DEX-induced increase in phosphorylated PDK1 levels, although the expression level of PDK1 was not affected (Figure 3I). Stimulating HEK293 cells with DEX elevated both SGK1 expression and phosphorylation (Figure 3J). On the other hand, pretreatment with WORT also inhibited the DEX-induced increase in SGK1 phosphorylation, but failed to inhibit the DEX-induced upregulation of SGK1 expression (Figure 3J).

These results show that in the oligodendrocytes of the corpus callosum, (1) repeated exposure to WIRS increases plasma corticosterone levels, (2) increased plasma corticosterone levels induce PDK1 phosphorylation, (3) upregulated phosphorylated PDK1 in turn phosphorylates SGK1, and (4) the increase in SGK1 expression is not induced by the activation of the PI3K pathway.

### Upregulation of phosphorylated SGK1 in oligodendrocytes increases NDRG1 phosphorylation

**Figure 5:**
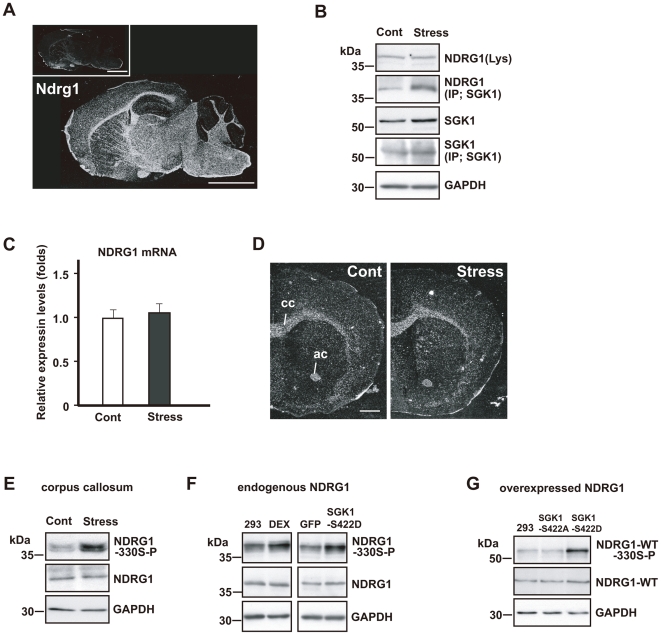
Repeated exposure to WIRS upregulates NDRG1 phosphorylation in the fiber tracts *via* SGK1 activation. (A) *In situ* hybridization images of *Ndrg1* mRNA. Dark-field photomicrographs show the distribution of *Ndrg1* mRNA-expressing cells in the mouse brain on the sagittal plane. Sections were hybridized with ^35^S-labeled antisense RNA probe for *Ndrg1* mRNA. As controls, adjacent sections were hybridized with ^35^S-labeled sense RNA probe (inset). Scale bar = 5 mm. (B) Immunoprecipitation and western blot analysis show that repeated exposure to WIRS elevated the interaction between SGK1 and NDRG1 (second column). However, NDRG1 expression did not increase in the corpus callosum (first column). (C, D) Real-time PCR analysis (C) and *in situ* hybridization histochemistry (D) show no alterations in *Ndrg1* mRNA levels in the corpus callosum of mice exposed to repeated WIRS (see also first panel of B). cc, corpus callosum; ac, anterior commissure. Scale bar = 2 mm. (E) Western blot analysis shows that repeated exposure to WIRS elevated phosphorylated NDRG1 levels in the corpus callosum. (F) Western blot analysis shows that DEX stimulation for 24 h elevated phosphorylated NDRG1 levels in HEK293 cells (left panels). The active form of SGK1 (SGK1-S422D) or control vector (GFP) were overexpressed in HEK293 cells for 3 days (right panels). Western blot analysis shows that SGK1-S422D overexpressed cells elevated phosphorylated NDRG1 levels. 293; no-stimulation control (HEK293 cells), DEX; 100 µM DEX for 24 h. (G) The negative form of SGK1 (SGK1-S422A) or the active form of SGK1 (SGK1-S422D) were overexpressed in the HEK293 cells expressing wild type NDRG1 (NDRG1-WT) for 3 days. Western blot analysis shows that the active form of SGK1 upregulates the phosphorylation level of the overexpressed wild type NDRG1.

Next, we attempted to elucidate the downstream target of SGK1. We examined the effects of increased phosphorylated SGK1 expression on NDRG1, because both *Sgk1* and *Ndrg1* are known to be expressed in the oligodendrocytes (Figure 5A) [17], [18], and NDRG1 is the substrate of SGK1 [19].

As mentioned above, repeated exposure to WIRS increased SGK1 expression levels in the corpus callosum (Figure 5B). However, the expression level of NDRG1 was not altered at either the protein (Figure 5B) or the mRNA level (Figure 5C, D) after repeated exposure to WIRS. Next, we examined the effect of repeated exposure to WIRS on the interaction between SGK1 and NDRG1 in the corpus callosum. We prepared immunoprecipitates by using a SGK1 antibody, and immunoblotted them with a NDRG1 antibody. Western blotting with a NDRG1 antibody by using lysates from the corpora callosa of control mice showed that NDRG1 co-immunoprecipitates with SGK1 (Figure 5B, second column, Cont). In addition, the amount of immunoprecipitate increased in the corpus callosum lysates after repeated exposure to WIRS (Figure 5B, second column, Stress). These findings strongly suggest that an increase in the level of phosphorylated SGK1 leads to stronger interactions between SGK1 and NDGR1, which, in turn, results in NDRG1 phosphorylation; this is because SGK1 is a kinase, which phosphorylates their substrates [20], [21]. Consequently, we examined this in further detail in the next experiment.

It has been demonstrated that S-330 in the NDRG1 protein is an important site phosphorylated by SGK1 [19], [22]. Therefore, we studied the effects of repeated exposure to WIRS on the phosphorylation of NDRG1 S-330. The phosphorylation level of NDRG1 in the corpus callosum was markedly elevated after repeated exposure to WIRS, although its protein expression level was not altered (Figure 5E). This finding indicates that the increased expression of phosphorylated SGK1 induced by chronic stress results in the phosphorylation of NDRG1 in the oligodendrocytes.

Next, we attempted to confirm this observation in vitro by using HEK293 cells. As mentioned above (i.e., the in vivo experiment), DEX treatment also increased the expression of phosphorylated SGK1 in vitro (Figure 3J). Furthermore, stimulating HEK293 cells with DEX increased the levels of phosphorylated NDRG1 and SGK1; this was achieved by overexpression of the constitutively active form of SGK1 (SGK1-S422D), which resulted in the phosphorylation of endogenous NDRG1 (Figure 5F). On the other hand, the overexpression of an inactive form of SGK1 (SGK1-S422A) failed to phosphorylate NDRG1 (data not shown). Overexpression of SGK1-S422D but not SGK1-S422A also increased the phosphorylation of overexpressed NDRG1 (Figure 5G).

### Up-regulation of SGK1 phosphorylation and the subsequent increase of NDRG1 phosphorylation following repeated exposure to WIRS upregulate the expression of adhesion molecules in oligodendrocytes

It has been demonstrated that NDRG1 plays a key role in stabilizing the adherens junctions by upregulating the recycling of E-cadherin in prostate cancer cells [23], [24]. Therefore, we examined the change in the expression levels of several adhesion molecules of the adherens junction in the corpus callosum after repeated exposure to WIRS.

**Figure 6:**
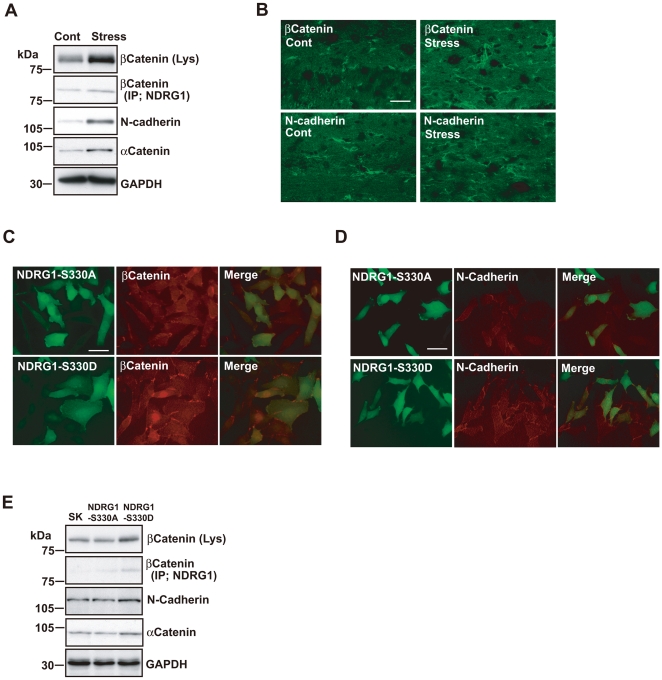
Repeated exposure to WIRS upregulates adhesion molecules expression levels in oligodendrocytes of the fiber tracts. (A) Immunoprecipitation and western blot analysis show that repeated exposure to WIRS elevated the interaction between NDRG1 and β-catenin (second panel), and that the expression levels of β-catenin, *N*-cadherin, and α-catenin were elevated in the corpus callosum. (B) Immunohistochemical analysis of β-catenin and *N*-cadherin in the corpus callosum demonstrates increased labeling of the processes of the oligodendrocytes (i.e., greater number and intensity) in mice exposed to repeated WIRS. Scale bar = 50 µm. (C, D) Immunocytochemical analysis of β-catenin and *N*-cadherin in SK-N-SH cells overexpressing non-phosphorylated (NDRG1-S330A) or phosphorylated NDRG1 (NDRG1-S330D). Increased expression of β-catenin (C) and *N*-cadherin (D) due to the overexpression of the phosphorylated form of NDRG1 (NDRG1-S330D) were mostly observed on the surfaces of the cell bodies and the processes of SK-N-SH cells. Scale bar = 20 µm. (E) Immunoprecipitation and western blot analysis show that overexpression of phosphorylated NDRG1 (NDRG1-S330D) upregulated the interaction between NDRG1 and β-catenin (second column), and that the expression levels of β-catenin, *N*-cadherin, and α-catenin were elevated in SK-N-SH cells. SK, no-transfection control (SK-N-SH cells).

We found that the expression levels of the main adhesion molecules, such as N-cadherin, α-catenin, and β-catenin, which comprise adherens junctions, increased in the corpus callosum after repeated exposure to WIRS (Figure 6A). This result was confirmed by subsequent immunohistochemical analysis, indicating that these molecules are localized in the oligodendrocytes of the corpus callosum (Figure 6B). The processes of the oligodendrocytes labeled by these molecules and found in the corpus callosum after repeated exposure to WIRS were more numerous and thicker than those of the controls. Furthermore, these processes could be traced for longer distances than those of the controls (Figure 6B).

Next, we determined if these adhesion molecules interact with NDRG1. Immunoprecipitation with an NDRG1 antibody by using lysates of the oligodendrocytes of the corpus callosum of normal mice showed that NDRG1 co-immunoprecipitates with β-catenin but not with *N*-cadherin or α-catenin. In addition, the co-immunoprecipitation of NDRG1 with β-catenin increased in the lysates of the corpus callosum after repeated exposure to WIRS (Figure 6A, second column).

To investigate the function of the increases in these adhesion molecules after repeated exposure to WIRS, we compared the subcellular localizations of these adhesion molecules between SK-N-SH cells expressing phosphorylated (NDRG1-S330D) and non-phosphorylated (NDRG1-S330A) NDRG1. In the cells expressing NDRG1-S330A, weak diffusely distributed staining was observed for each of these adhesion molecules throughout the cytoplasm, including the cell membrane (Figure 6C, 6D, upper-middle panel). In the cells expressing NDRG1-S330D, more pronounced immunoreactivity was detected for each of the three adhesion molecules at the membrane surface (Figure 6C, 6D, lower-middle panel). Although the overexpression of NDRG1-S330D increased the expression of *N*-cadherin, α-catenin, and β-catenin, overexpression of NDRG1-S330A had no noticeable effect on the expression levels of these molecules (Figure 6E). In addition, the co-immunoprecipitation of NDRG1 with β-catenin increased after NDRG1-S330D overexpression (Figure 6E). These findings demonstrate that (1) repeated exposure to WIRS increases the expression of phosphorylated NDRG1 and the interaction between phosphorylated NDRG1 and β-catenin; (2) increased phosphorylated NDRG1 expression upregulates the expression of *N*-cadherin, α-catenin, and β-catenin; and (3) increased amounts of these adhesion molecules are concentrated at the cell membrane.

### Repeated exposure to WIRS causes morphological alterations to oligodendrocytes in the corpus callosum

The above findings show that repeated exposure to WIRS increases the expression of *N*-cadherin, α-catenin, and β-catenin in the oligodendrocytes. In addition, as shown in Figure 6B, the processes of the oligodendrocytes expressed in the corpus callosum after repeated exposure to WIRS were much more numerous and thicker, and could be traced over longer distances than those of the controls. This suggests that the processes of these oligodendrocytes cause the branching observed after repeated exposure to WIRS. Therefore, we subsequently studied the morphological changes in the corpus callosum after repeated exposure to WIRS.

**Figure 7:**
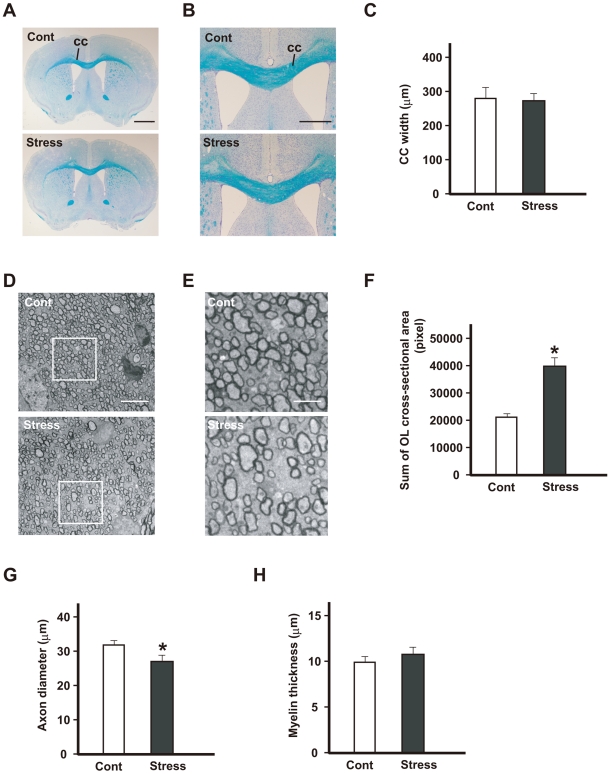
Repeated exposure to WIRS causes morphological alterations in oligodendrocytes in the corpus callosum. (A, B): Representative histological coronal sections through the forebrain of control (Cont) and repeated WIRS-exposed mice (stress) with Kluver–Barrera stain. No distinct changes were detected in the corpus callosum (cc) between the 2 groups of mice at either lower (A) or higher (B) magnification. Scale bar = 2 mm in (A) and 1 mm in (B). (C) Results of the quantification of the width of the corpus callosum. Results are expressed as the mean ± SEM of 3 independent experiments. (D, E) Representative transverse electron micrographs of the corpus callosum from control (upper panels of D and E) and repeated WIRS-exposed mice (lower panels of D and E). Scale bars = 5 µm. (E) The higher magnification of the square region of (D). Scale bar = 2 µm. (F) Results of the quantification of the sum of oligodendrocytes in the cross-sectional area. The results are expressed as the mean ± SEM of 3 independent experiments. *p<0.05, *t*-test. (G, H) The distributions of the axon diameters (G) and myelin thicknesses (H) of the corpus callosum in the control and repeated WIRS-exposed mice were assessed. The results are expressed as the mean ± SEM of 3 independent experiments. *p<0.05, *t*-test.

Light microscopic observations using Kluver–Barrera staining revealed no clear alterations in the corpus callosum with respect to thickness; a travelling pattern was clearly identifiable after repeated exposure to WIRS (Figure 7A–C). However, the electron microscopy analysis clearly showed morphological changes in the oligodendrocytes in the corpus callosum after repeated exposure to WIRS (Figure 7D–F). In the control mice, a number of myelinated fibers were compactly gathered in the corpus callosum (Figure 7D, E, Cont). However, the interfibral space, which is occupied almost entirely by the oligodendrocytes, increased markedly in the corpus callosum following repeated exposure to WIRS (Figure 7D, E, Stress). Statistical analysis shows that the interfibral space in WIRS-exposed mice was twice as large as that in the control mice (Figure 7F). In addition, the average diameter of the nerve fibers was smaller in the corpus callosum of WIRS-exposed mice than those of the controls (Figure 7G). However, there was no significant change in the thickness of the myelin in the corpus callosum of the WIRS-exposed mice compared to the controls (Figure 7H). Thus, these findings demonstrate that repeated exposure to WIRS causes excess arborization of oligodendrocyte processes. However, it is not clear whether the PDKI-SGK1-NDRG1-adhesion molecules pathway or another unknown system activated by repeated exposure to WIRS induces the excess arborization of their processes. Therefore, we addressed this question in the following experiments.

### Activation of the PDKI-SGK1-NDRG1 pathway by repeated exposure to WIRS increases oligodendrocyte volume in the corpus callosum

We investigated this hypothesis by using cultured mature oligodendrocytes and immature oligodendrocytes. We first discriminated mature from immature oligodendrocytes by using two different markers of oligodendrocytes: NG2 was used as a marker of immature oligodendrocytes (OPCs; oligodendrocyte precursor cells) and the myelin basic protein (MBP) for mature oligodendrocytes [25], [26]. In addition, since oligodendrocytes change their shape during differentiation *in vitro*, their morphology is also a good guide for evaluating the developmental stage; mature oligodendrocytes have numerous processes and arborizations, and oligodendrocytes have longer processes than OPCs (Figure S2).

**Figure 8:**
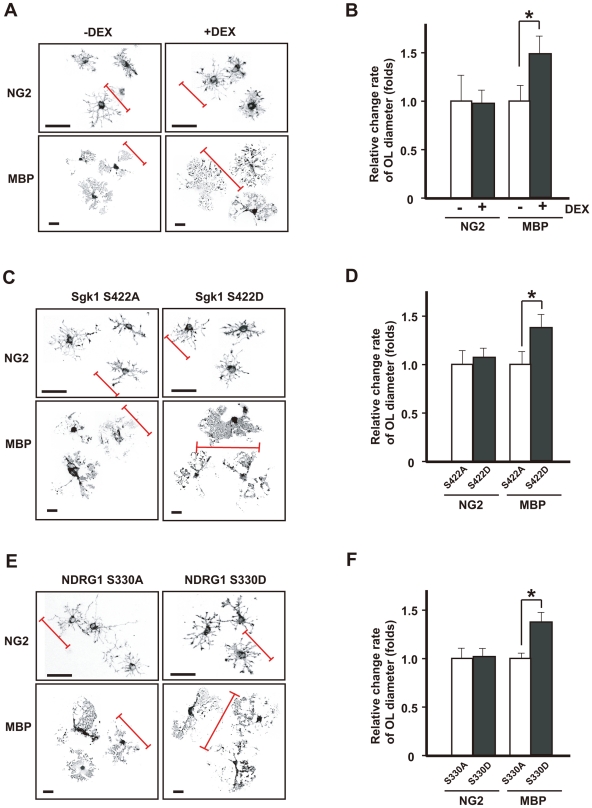
Activation of the SGK1-NDRG1 pathway also causes morphological changes in primary cultured oligodendrocytes. (A, C, E) Morphology of primary cultured oligodendrocytes treated with (+DEX) (a, right column) or without DEX (-DEX) for 2 days (A, left column). Overexpression of the active form of SGK1 (SGK1-S422D) (C, right column) and overexpression of phosphorylated NDRG1 (NDRG1-S330D) (E, right column) by using the ImageJ tracing tool. As controls for the last 2 experiments, the effects of the overexpression of SGK1-S422A (the inactive form of SGK1) (C, left column) and NDRG1-S330A (the non-phosphorylated form of NDRG1) (E, left column) were examined. NG2 and MBP were used as markers of immature and mature oligodendrocytes, respectively. (B, D, F) Quantification of oligodendrocyte size (diameters) is shown in panels A, C, and E, respectively. Morphometric measurements of oligodendrocyte diameters were performed using ImageJ software. The results are expressed as the mean ± SEM of 3 independent experiments. *p<0.05, *t*-test.

The addition of DEX resulted in an approximately 1.5-fold increase in the diameter of the oligodendrocytes labeled by MBP, compared to the untreated oligodendrocytes (control) (Figure 8A, B). Overexpression of the active forms of SGK1 (SGK1-S422D) and NRDG1 (NDRG1-S330D) in the oligodendrocytes had the same effect as repeated exposure to WIRS. Overexpression of the inactive forms of SGK1 (SGK1-S422A) and NDRG1 (NDRG1-S330A) did not induce morphological changes in oligodendrocytes, while overexpression of SGK1-S422D and NDRG1-S330D increased the diameter of the oligodendrocytes (1.38- and 1.39-fold increases for SGK1 and NDRG1, respectively) (Figure 8C–F).

However, the effects of DEX as well as of active SGK1 and NDRG1 on oligodendrocytes could not be observed in the OPCs labeled with NG2 (Figure 8). These results indicate that the HPA axis–SGK1-NDRG1 signaling pathway strongly impacts oligodendrocytes causing morphological alterations, whereas this is not observed in OPCs.

**Figure 9:**
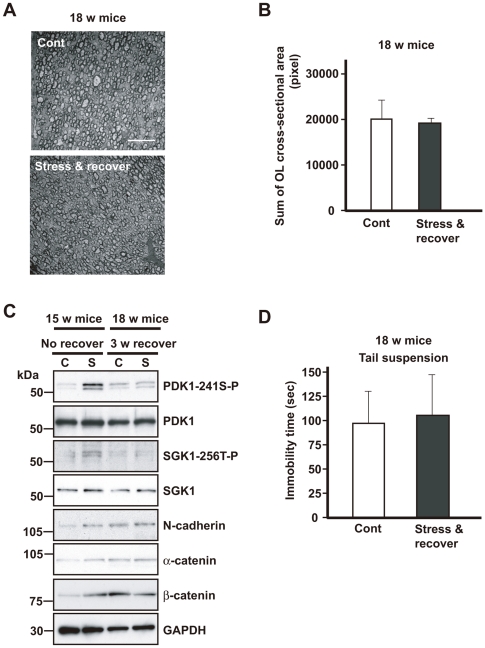
Activation of the PDKI-SGKI-NDRG1-adhesion molecule pathway returns to the control level after 3 weeks recovery. (A) Representative transverse electron micrographs of the corpus callosum of no-stress controls (18-week-old mice) (Cont) and after 3 weeks recovery after exposure to WIRS (18-week-old mice) (stress & recover). Scale bars = 10 µm. (B) Quantification of the sum of the cross-sectional areas of the oligodendrocytes is shown in panel A. Morphometric measurements were made using ImageJ software. The results are expressed as the mean ± SEM of 3 independent experiments. *p<0.05, *t*-test. (C) Western blot analysis of the corpora callosa of mice exposed to repeated WIRS (15-week-old mice) and after 3 weeks recovery (18-week-old mice). In the lysates from the corpora callosa of the mice in which repeated exposure to WIRS was discontinued, no increase in PDK1 phosphorylation, SGK1 expression or phosphorylation, or the expression of adhesion molecules such as β-catenin was identified. (D) Effects of a chronic stress, i.e., 3-week recovery after exposure to repeated WIRS, on mouse behavior. Mice in which repeated exposure to WIRS (18-week-old mice) was discontinued show no significant difference in the tail-suspension test compared to the control mice (18-week-old mice). The results are expressed as the mean ± SEM of 3 independent experiments. *p<0.05, *t*-test.

Finally, it should be noted that the increase in the immobility time and interfibral space in the corpus callosum following repeated exposure to WIRS returned to control levels after interrupting exposure to WIRS for 3 weeks (Figure 9A, B, and D). Furthermore, the activation of the PDK1–SGK1–NDRG1–adhesion molecules pathway following repeated exposure to WIRS did not differ between groups following repeated exposure to WIRS (chronic stress) (S) and control mice (C) after interrupting exposure to WIRS for 3 weeks (Figure 9C, 18 w mice).

## Discussion

This study demonstrates for the first time both in vivo and in vitro that (1) repeated exposure to WIRS activates PDKI–SGK1–NDRG1–adhesion molecules (i.e., *N*-cadherin, α-catenin, and β-catenin) expression pathway *via* an increase in plasma corticosterone levels, (2) the activation of this signaling pathway causes excess arborization of oligodendrocyte processes, and (3) this abnormality in the oligodendrocytes is related to depression-like symptoms. This is because the abnormal arborization of oligodendrocytes and depression-like symptoms returned to the control levels after mice recovered from the chronic stress (Figure 9, Figure S3).

Both simple and repeated WIRS resulted in the upregulation of plasma corticosterone levels *via* the activation of the HPA axis [27], [28]. It was previously reported that subcutaneous injection of corticosterone causes the upregulation of SGK1 in oligodendrocytes [29], suggesting that various stressors that induce increases in plasma corticosterone levels possibly upregulate SGK1 expression in oligodendrocytes. The present study reveals that WIRS not only increases SGK1 expression, but also the level of phosphorylated SGK1 (the active form of SGK1). Since the WIRS-induced increase in Sgk1 expression in the oligodendrocytes was abolished in adrenalectomized mice (Figure 1), Sgk1 expression in the oligodendrocytes is induced by the elevation of plasma corticosterone levels *via* the activation of the HPA axis.

The upregulation of Sgk1 in oligodendrocytes after cortical injury was first reported by Imaizumi et al. [30], followed by hippocampal injury by Hollister et al. [31]. Since brain injury does not induce the elevation of plasma corticosterone levels, a mechanism other than the activation of the HPA pathway is involved in the upregulation of Sgk1 after brain injury.

In addition to oligodendrocytes, it has been shown that Sgk1 or SGK1 is expressed in the neurons in several diseases such as Parkinson's disease [32]–[34], Huntington's disease [35], amyotrophic lateral sclerosis (ALS) [34], and ischemia [36]. Moreover, in experimental animals, the administration of morphine [37], tumor necrosis factor (TNF)-α, or transforming growth factor (TGF)-β [38], [39], [40], [41] also upregulate Sgk1 expression in neurons. However, the molecular mechanism underlying the elevation of Sgk1 in neurons remains unknown.

Several molecules that interact with SGK1 in the brain are reported. Among them, NDRG1 has been shown to be localized in oligodendrocytes [18]. Here, we confirmed that SGK1 and NDRG1 also interact in the oligodendrocytes. Furthermore, we showed that repeated exposure to WIRS and phosphorylated SGK1 increase the phosphorylation of NDRG1, although these have no effects on the NDRG1 expression level (Figure 5).

NDRG1 has been shown to play a key role in stabilizing adherens junctions by upregulating recycled E-cadherin in prostate cancer cells [23], [24]. In this study, we examined changes in the expression levels of several adhesion molecules related to the adherens junction in the corpus callosum after repeated exposure to WIRS. We found that the expression of *N*-cadherin, α-catenin, and β-catenin increased in the corpus callosum after repeated exposure to WIRS (Figure 6). In addition, we detected interaction between NDRG1 and β-catenin but not *N*-cadherin or α-catenin (Figure 6).

It is reported that SGK1 is a cell volume-sensitive gene [38], [42]. Therefore, we investigated whether the activation of the PDK1–SGK1–NDRG1 pathway increases the volume of oligodendrocytes, or if the increase in SGK1 expression is a passive event resulting from the enlarged oligodendrocytes after repeated exposure to WIRS. We found that the overexpression of the phosphorylated forms of SGK1 and NDRG1 increased the size of oligodendrocytes (Figure 8). Therefore, it can be concluded that the activation of the PDK1–SGK1–NDRG1 pathway by repeated exposure to WIRS directly induces the increase in the cellular volume of oligodendrocytes.

However, the functional significance of the enlargement of oligodendrocytes occupying the intrafibrial space in the corpus callosum is not clear. Thus, we examined the change in MBP levels in the corpora callosa of the mice exposed to repeated WIRS, and found that the expression of MBP was markedly reduced in mice exposed to chronic stress (Figure 4). Since phosphorylated MBP is actively expressed during the early stage of myelin formation [43], it is likely that after repeated exposure to WIRS, impaired myelination of oligodendrocytes in the corpus callosum leads to the increase in the cytoplasmic volume of oligodendrocytes. MBP expression is also reported to decrease in the prefrontal cortex in a social stress model of rats [44]. In any case, abnormal swelling of the cytoplasm in oligodendrocytes may impair neurotransmission or interactions among the nerve fibers in the corpus callosum [45], [46].

Several recent studies report that major depression impairs oligodendrocyte function through decreased MBP expression [47] or reduced corpus callosum size in female depression patients [48] or low densities of total glia and oligodendrocytes in the amygdala [49], or reduced expression of oligodendrocyte-related genes in the temporal cortex [50]. The present study shows that elevation of plasma corticosterone levels by repeated exposure to WIRS induces the expressions of adhesion molecules in oligodendrocytes *via* the activation of the PI3K–PDK1–SGK1–NDRG1 pathway, which causes excess arborization of oligodendrocytes. However, no reduction in the number of oligodendrocytes was detected in the corpus callosum. Elucidating the functional roles of the PI3K–PDK1–SGK1–NDRG1 pathway in the corpus callosum is a primary goal of future research.

## Supporting Information

Figure S1 **Neuronal Sgk1 expression was not affected by acute stress.** (A) Sgk1mRNA is expressed in the neurons of CA3 of the hippocampus of the control mice (Cont). However, no increase in Sgk1 mRNA expression was detected in these neurons after repeated WIRS (Stress). (B) Quantification of the results is shown in panel a. These hippocampus Sgk1 mRNA intensities were calculated by using ImageJ software.
(TIF)

Figure S2 **Morphology of primary oligodendrocytes.** Morphology of immature (NG2, red) and mature (MBP, green) primary cultured oligodendrocytes after 4 days of differentiation induction.
(TIF)

Figure S3 **Elevation of corticosterone induced by repeated WIRS induce the adherent molecules and morphological change in the oligodendrocytes of corpus callosum via the activation of PDK1-SGK1-NDRG1 pathway.**
(TIF)

Materials and Methods S1 (DOC)
