# Supplementary figures and images for: Plasma Corticosterone Activates SGK1 and Induces Morphological Changes in Oligodendrocytes in Corpus Callosum
Source: PLoS One. 2011 May 31;6(5):e19859. doi: 10.1371/journal.pone.0019859 (PMC3104997; doi:10.1371/journal.pone.0019859)

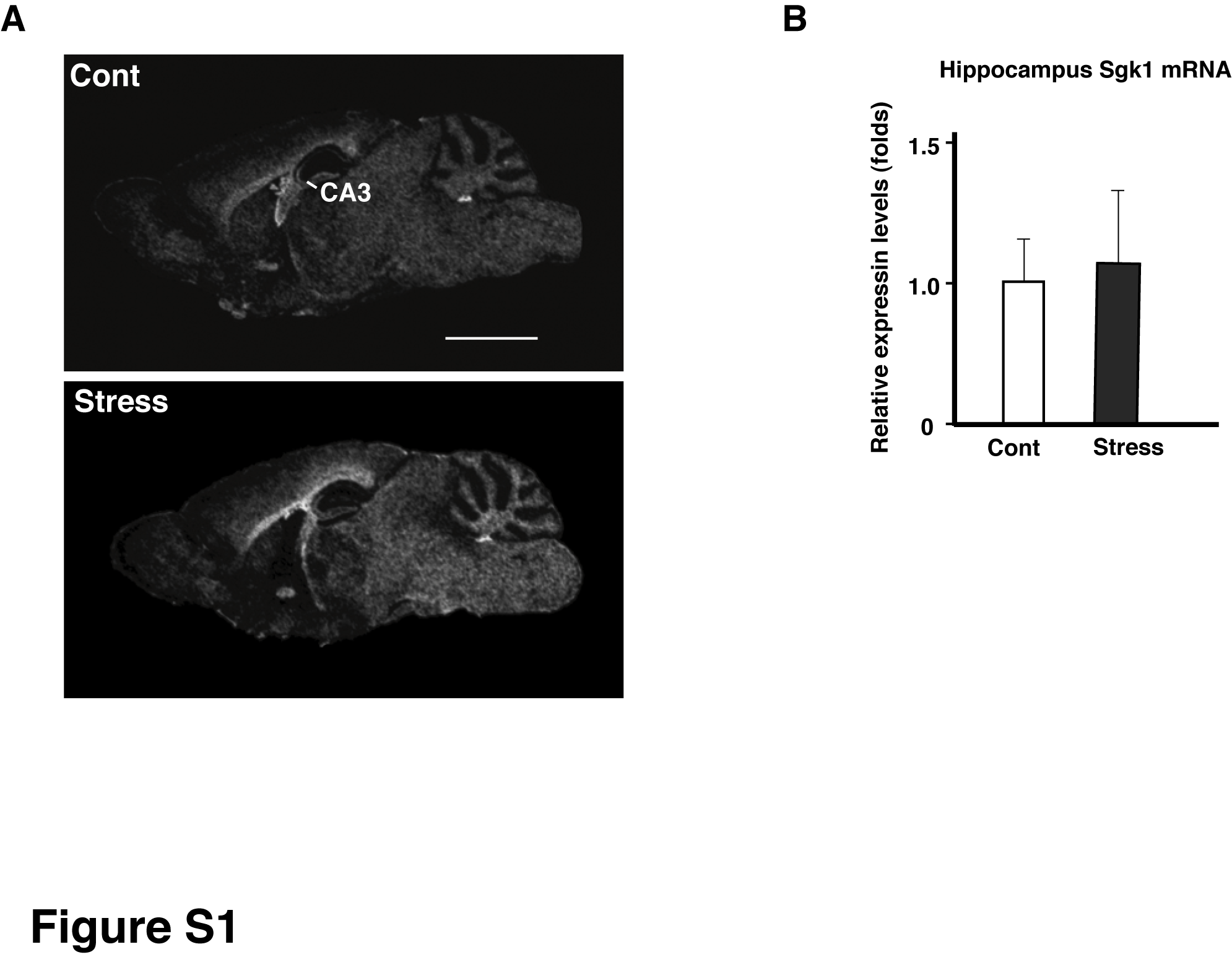

Supplement: Figure S1 — Neuronal Sgk1 expression was not affected by acute stress. (A) Sgk1mRNA is expressed in the neurons of CA3 of the hippocampus of the control mice (Cont). However, no increase in Sgk1 mRNA expression was detected in these neurons after repeated WIRS (Stress). (B) Quantification of the results is shown in panel a. These hippocampus Sgk1 mRNA intensities were calculated by using ImageJ software. (TIF) [file pone.0019859.s001.tif]

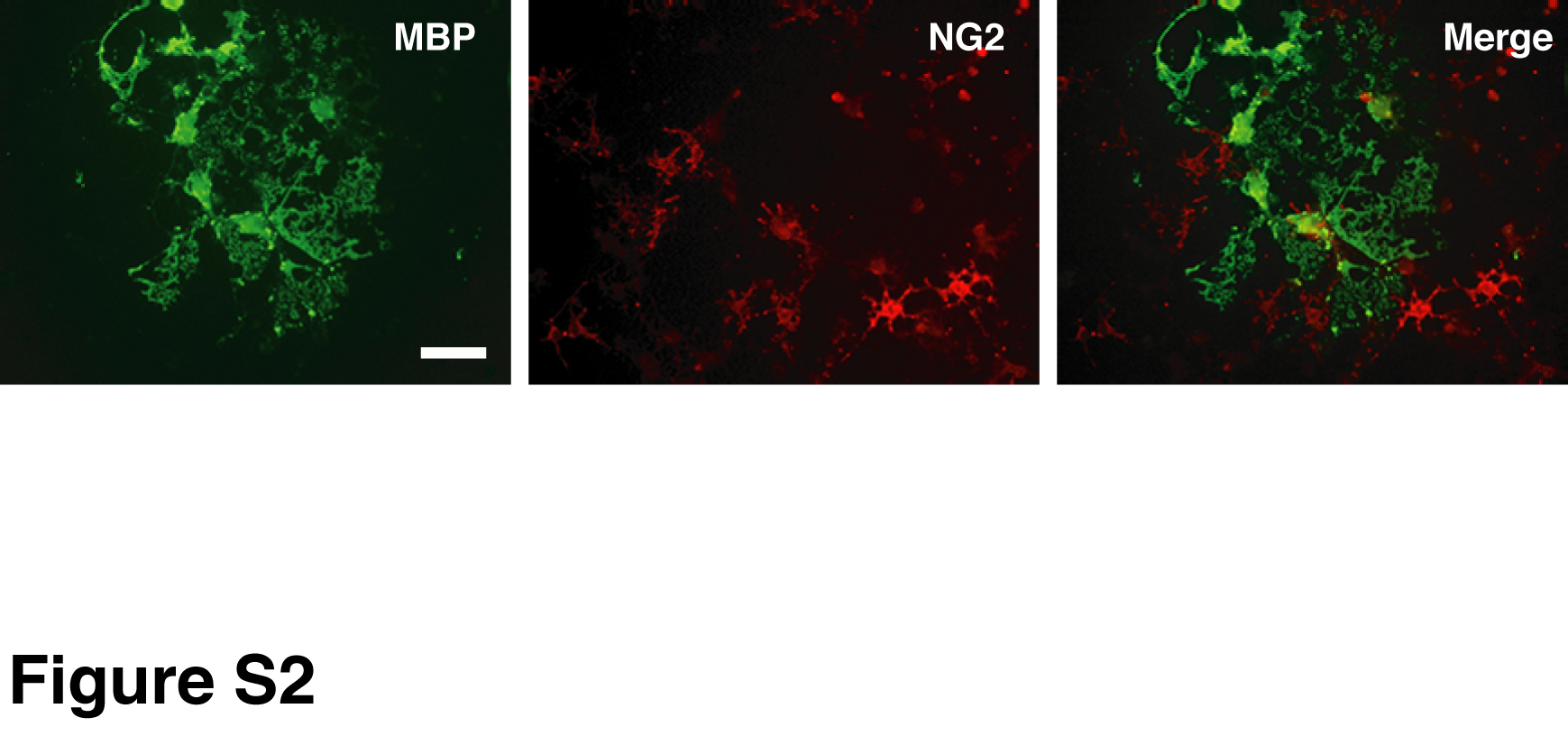

Supplement: Figure S2 — Morphology of primary oligodendrocytes. Morphology of immature (NG2, red) and mature (MBP, green) primary cultured oligodendrocytes after 4 days of differentiation induction. (TIF) [file pone.0019859.s002.tif]

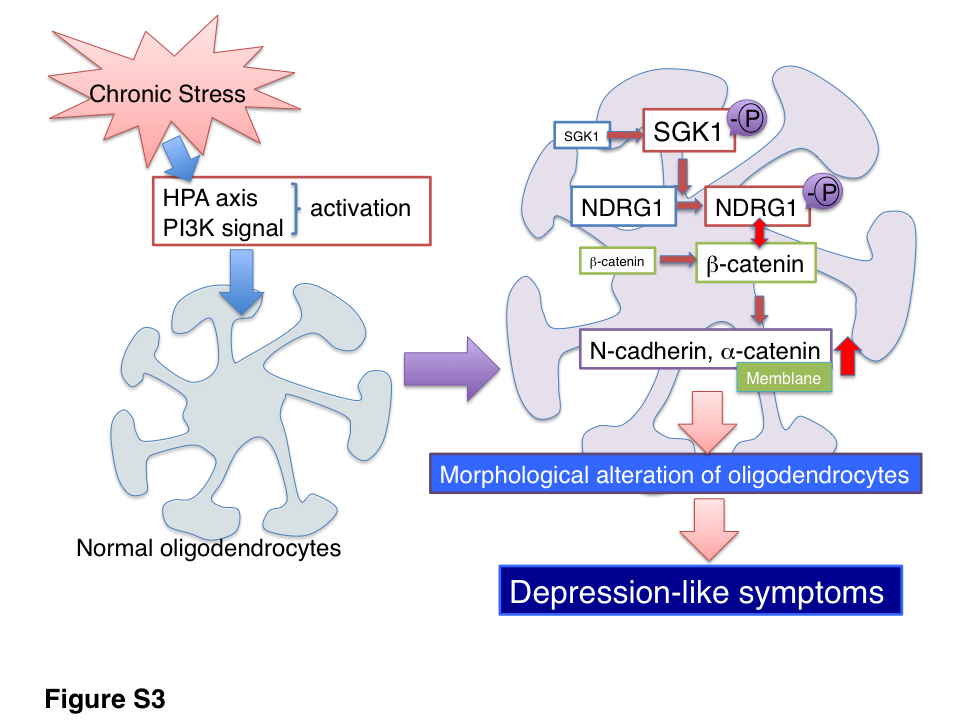

Supplement: Figure S3 — Elevation of corticosterone induced by repeated WIRS induce the adherent molecules and morphological change in the oligodendrocytes of corpus callosum via the activation of PDK1-SGK1-NDRG1 pathway. (TIF) [file pone.0019859.s003.tif]
