# Supplementary material for: Plasma Corticosterone Activates SGK1 and Induces Morphological Changes in Oligodendrocytes in Corpus Callosum
Source: PLoS One. 2011 May 31;6(5):e19859. doi: 10.1371/journal.pone.0019859 (PMC3104997; doi:10.1371/journal.pone.0019859)
Supplement: Materials and Methods S1 — (DOC) [file pone.0019859.s004.doc]

**Supplemental Information**

**Materials and Methods S1**

***Western blot analysis***

Western blot analysis was performed as previously described (Ikenaka et al., 2006). We used the anti-SGK1 antibody (1:1000) (Abcam Inc., MA, USA), anti-NDRG1 antibody (1:500) (Santa Cruz Biotechnology, Santa Cruz, CA USA), anti-GAPDH antibody (1:10000) (Santa Cruz Biotechnology), anti-phospho-SGK1 (T256) antibody (1:500) (Cell signaling, MA, USA), anti-phospho-SGK1 (S422) antibody (1:500) (Abcam Inc., MA, USA), anti-phospho-NDRG1 (S330) antibody (1:500) (Cell signaling), anti-PDK1 antibody (1:500) (Cell signaling), anti-phospho-PDK1 (S241) antibody (1:500) (Cell signaling), anti-N-cadherin antibody (1:500) (BD bioscience, NJ, USA), anti-α-catenin antibody (1:500) (BD bioscience), anti-β-catenin antibody (1:500) (BD bioscience), anti-NG2 antibody (1:1000) (Millipore, Billerica, MA, USA) and anti-MBP antibody (1:500) (Millipore). Immunodetection was performed using the ECL Western Blotting Detection System (GE Healthcare) with peroxidase-coupled secondary antibodies according to the manufacturer’s instructions.

***Plasmid construction***

A green fluorescent protein (GFP)-fused SGK1 or NDRG1 plasmids with EF-1α promoter were constructed by using the pENTR/D-TOPO vector (Invitrogen Corp.). The EF-1a promoter was amplified from pEF5/FRT/V5-D-TOPO vector by using the following primer pair: 5′-GCCGCCCCCTTCACCGGAGTGCCTCGTGAGGCTCCGGTG-3′ (forward) and 5′-GGCGCGCCCACCCTTAGTACTTCACGACACCTGAAATGGAAGAAA-3′ (reverse). The GFP moiety was amplified from ppcDNA6.2-GW/EmGFP-miR vector (Invitrogen Corp.) by using the following primer pair: 5′-CAGGTGTCGTGAAGTGCCACCATGGTGAGCAAGGGCGAGGAGCTG-3′ (forward) and 5′-GCGCCCACCCTTAGTCTCTAGATCAACCACTTTGTACAA-3′ (reverse). Wild type mouse *Sgk1* (∆N-SGK1) and *Ndrg1* were cloned using a PCR-based method for the eukaryotic expression system. *Sgk1* and *Ndrg1* were amplified by PrimeSTAR® Max DNA Polymerase (Takara Bio Inc., Ohtsu, Japan) using a primer set; *Sgk1* (∆N-SGK1); 5′-TCGTGAAGTGCCACCATGTCCCATCCTCAGGAGCCGGAG-3′ (forward) and 5′-CTCCTCGCCCTTGCTCACGAGGAAGGAATCCACAGGAGG-3′ (reverse), *Ndrg1*; 5′-TCGTGAAGTGCCACCATGTCCCGAGAGCTACATGACGTG-3′ (forward) and 5′-CTCCTCGCCCTTGCTCACGCAGGACACCTCCATGGACTT-3′ (reverse). The template was mouse brain cDNA library (Clontech, Mountain Veiw, CA, USA). The amplified fragments were cloned into the pENTR/D-TOPO vector by using In-Fusion Advantage PCR Cloning Kit (Takara Bio Inc.) according to the manufacturer’s instructions. For the SGK1 constitutive active or negative form (CA or CN) constructs, SGK1-S422D (CA) or -S422A (CN) were amplified from pENTR-SGK1-GFP by using the following primer sets: SGK1-S422D; 5′-GCCTTCCTCGGCTTCgaCTATGCACCTCCTGTG-3′ (forward) (The mutated sequences are indicated in lowercase letters; these mutations change the amino acid sequence of mouse SGK1 from S422 to D422) and 5′-TTCTGCTGCTTCCTTCACACTGGC-3′ (reverse), SGK1-S422A; 5′-GCCTTCCTCGGCTTCgCCTATGCACCTCCTGTG-3′ (forward) (The mutated sequences are indicated in lowercase letters; these mutations change the amino acid sequence of mouse SGK1 from S422 to A422) and 5′-TTCTGCTGCTTCCTTCACACTGGC-3′ (reverse). For the NDRG1 constitutive phosphorylate or nonphosphorylate form (S330D or S330A) constructs, NDRG1-S330D or –S330A were amplified from pENTR-NDRG1-GFP by using the following primer sets: NDRG1-S330D; 5′-CGGTCCCGCACAGCCgaTGGCTCCAGTGTCACA-3′ (forward) (The mutated sequences are indicated in lowercase letters; these mutations change the amino acid sequence of mouse NDRG1 from S330 to D330) and 5′-CATCAGGCGAGTCATGCTGGCAGA-3′ (reverse), NDRG1-S330A; 5′- CGGTCCCGCACAGCCgCTGGCTCCAGTGTCACA-3′ (forward) (The mutated sequences are indicated in lowercase letters; these mutations change the amino acid sequence of mouse NDRG1 from S330 to A330) and 5′-CATCAGGCGAGTCATGCTGGCAGA-3′ (reverse). A recombinant adenovirus expressing GFP, GFP fused mouse SGK1, GFP fused mouse NDRG1 were generated using the ViraPower Adenoviral Expression system (Invitrogen Corp.) according to the manufacturer's instructions.

***Cell culture***

HEK293 cells were maintained in tissue-culture dishes (Nunc) with DMEM containing 10% heat-inactivated fetal bovine serum (FBS). SK-H-SH cells were maintained in tissue-culture dishes (Nunc) with αMEM containing 10% heat-inactivated FBS at 37°C in an atmosphere of 95% air/5% CO_2_. The HEK293 and SK-H-SH cells were transfected by using Lipofectamine 2000 or Lipofectamine RNAiMAX (Invitrogen Corp.) according to the manufacturer’s instructions.

Oligodendroglial cell cultures were prepared from P1 Wistar rat cortex by using a previously described method (Chen et al., 2007). Briefly the cells were spread on poly-L-lysine-coated flasks with αMEM containing 10% heat-inactivated FBS at 37°C in an atmosphere of 95% air/5% CO2 for 14 days. For isolation and culture of rat oligodendroglial cells, shake the flasks at 200 rpm for 12 hrs and the cells were spread on poly-L-lysine-coated flasks at a density of 1 × 10^4^ cells/cm^2^ with B27-supplement-, PDGF-, NT-3-, insulin-containing NEUROBASAL Medium (Invitrogen Corp.) for 3 days, and the oligodendroglial cells were changed for PDGF free NEUROBASAL Medium. The neurons were transfected by using Lipofectamine LTX and Plus regents or Lipofectamine RNAiMAX (Invitrogen Corp.) according to the manufacture’s instructions.

***Immunocytochemical procedure***

SK-N-SH cells and primary oligodendrocyte cells were grown on four-well chamber dishes at a density of 3 × 10^4^ cells/cm^2^. These cells were fixed in 4% paraformaldehyde in 0.1M PBS, and incubated at 4°C in a humid atmosphere for 1 day with an anti-GFP antibody, anti-N-cadherin antibody and anti-β-catenin antibody diluted in PBS containing 0.3% Triton X-100 (1:200) and 5% BSA. They were then rinsed with PBS for 1 hr and incubated at room temperature for 2 hrs with Alexa Fluor 488-conjugated goat anti-rabbit IgG antibody and Alexa Fluor 568-conjugated goat anti-mouse IgG antibody (Invitrogen Corp., Carlsbad, CA, USA) at a dilution of 1:500 in PBS. These cells were subsequently washed with PBS for 1 hr and mounted in PermaFluor. Confocal microscopy was performed using a Carl Zeiss LSM-510 confocal microscope equipped with 20× objective lenses. Morphometric measurements were made using ImageJ software.

***Immunohistochemical procedures***

The sections were incubated at 4°C in a humid atmosphere for 1 day with an anti-GFP antibody, anti-N-cadherin antibody and anti-β-catenin antibody diluted in PBS containing 0.3% Triton X-100 (1:200) and 5% BSA. They were then rinsed with PBS for 1 hr and incubated at room temperature for 2 hrs with Alexa Fluor 488-conjugated goat anti-rabbit IgG antibody and Alexa Fluor 568-conjugated goat anti-mouse IgG antibody (Invitrogen Corp., Carlsbad, CA, USA) at a dilution of 1:500 in PBS. The sections were subsequently washed with PBS for 1 hr and mounted in PermaFluor. Confocal microscopy was performed with a confocal microscope (LSM-510) with 20× objective lenses (Carl Zeiss, Oberkochen, Germany).
